# Systems modeling accurately predicts responses to genotoxic agents and their synergism with BCL-2 inhibitors in triple negative breast cancer cells

**DOI:** 10.1038/s41419-017-0039-y

**Published:** 2018-01-19

**Authors:** Federico Lucantoni, Andreas U. Lindner, Norma O’Donovan, Heiko Düssmann, Jochen H. M. Prehn

**Affiliations:** 1 Department of Physiology and Medical Physics, Royal College of Surgeons in Ireland, Dublin, 2 Ireland; 2 Centre for Systems Medicine, Royal College of Surgeons in Ireland, Dublin, 2 Ireland; 3 National Institute for Cellular Biotechnology, Dublin City University, Dublin, 9 Ireland

## Abstract

Triple negative breast cancer (TNBC) is an aggressive form of breast cancer which accounts for 15–20% of this disease and is currently treated with genotoxic chemotherapy. The BCL2 (B-cell lymphoma 2) family of proteins controls the process of mitochondrial outer membrane permeabilization (MOMP), which is required for the activation of the mitochondrial apoptosis pathway in response to genotoxic agents. We previously developed a deterministic systems model of BCL2 protein interactions, DR_MOMP that calculates the sensitivity of cells to undergo mitochondrial apoptosis. Here we determined whether DR_MOMP predicts responses of TNBC cells to genotoxic agents and the re-sensitization of resistant cells by BCL2 inhibitors. Using absolute protein levels of BAX, BAK, BCL2, BCL(X)L and MCL1 as input for DR_MOMP, we found a strong correlation between model predictions and responses of a panel of TNBC cells to 24 and 48 h cisplatin (*R*^2^ = 0.96 and 0.95, respectively) and paclitaxel treatments (*R*^2^ = 0.94 and 0.95, respectively). This outperformed single protein correlations (best performer BCL(X)L with *R*^2^ of 0.69 and 0.50 for cisplatin and paclitaxel treatments, respectively) and BCL2 proteins ratio (*R*^2^ of 0.50 for cisplatin and 0.49 for paclitaxel). Next we performed synergy studies using the BCL2 selective antagonist Venetoclax /ABT199, the BCL(X)L selective antagonist WEHI-539, or the MCL1 selective antagonist A-1210477 in combination with cisplatin. In silico predictions by DR_MOMP revealed substantial differences in treatment responses of BCL(X)L, BCL2 or MCL1 inhibitors combinations with cisplatin that were successfully validated in cell lines. Our findings provide evidence that DR_MOMP predicts responses of TNBC cells to genotoxic therapy, and can aid in the choice of the optimal BCL2 protein antagonist for combination treatments of resistant cells.

## Introduction

Breast cancer is a heterogeneous disease in which gene expression features and molecular classification has successfully helped in defining individualized therapies, leading to significant improvements in disease-specific survival^1,2^. Triple negative breast cancer (TNBC), a subset of breast cancer, is defined by the loss of estrogen receptor (ER), progesterone receptor (PgR), human epidermal growth factor receptor type 2 (HER2) expression and the presence of basal-like markers^3^. Treatment of this cancer is very challenging as responses are often poor and targeted therapies do not yet exist. Standard chemotherapeutic regimens for TNBC include drugs that induce DNA damage and elicit DNA repair mechanisms such as cisplatin and anthracyclines, microtubule stabilizing drugs such as taxanes, and antimetabolites such as 5-fluorouracil^4,5^. Patients with TNBC show variable responses to genotoxic chemotherapy^1,6,7^. Therefore there is a significant need to identify molecular biomarkers that predict patient responses to genotoxic chemotherapy, and to identify new suitable targeted therapies and associated patient stratification tools.

Genotoxic chemotherapy induces apoptosis in cancer cells. The BCL2 family of proteins are main regulators of the mitochondrial apoptosis pathway^8^. To date three different subfamilies have been identified based on structural and functional studies: the anti-apoptotic BCL2 subfamily, the pro-apoptotic BAX-like subfamily, and the pro-apoptotic BH3-only protein subfamily. BH3-only proteins are apoptosis initiators that are induced transcriptionally or activated post-translationally upon cytotoxic stress. BH3-only proteins either directly activate BAX and BAK, or inhibit the anti-apoptotic BCL2 proteins from binding their pro-apoptotic partners^9^. Upon activation BAX and BAK form pore structures in the mitochondrial outer membrane to release cytochrome-c and activate the apoptosome^10^. Additionally, BCL2 proteins have a physiological role in the regulation of mitochondrial bioenergetics and fusion/fission events^11^.

Given the central role of BCL2 family protein in tumor progression and therapy responses, several studies focused on their role as prognostic biomarkers in breast cancer^12^. Around 85% of ER positive breast cancer overexpress BCL2. In this subtype, high BCL2 levels are interestingly a prognostic marker for favorable outcome^13^. In TNBC, a co-amplification of MCL1 was found in 80% of cases harboring MYC amplification^14^. BCL2 was found to be an independent prognostic marker and to predict response to anthracycline combination chemotherapy in TNBC^15^. Furthermore, BCL(X)L has been described as a main driver in preventing cell death particularly in mesenchymal breast cancer cells^16^.

Due to the complexity and functional redundancies of the BCL2 interaction network, predictions of therapy responses based on single BCL2 protein expression levels are prone to be difficult. To address this shortfall, our group developed and validated a deterministic model of BCL2 protein interactions, DR_MOMP^17^. By taking into account expression levels as well as interaction kinetics of anti-apoptotic (BCL2, BCL(X)L, MCL1) and pro-apoptotic BCL2 proteins (BAX, BAK), the model calculates a stress dose, *η* (corresponding to the production rates of BH3-only proteins PUMA, NOXA, and BIM) that is required to induce MOMP in individual cells. It hence delivers a single value that indicates the cell’s sensitivity to mitochondrial apoptosis^17^. In a recent study our system model approach successfully identified high-risk stage III colorectal patients using frozen or fixed tissue samples, with the highest risk score among several molecular and pathological features^18^.

Recent years have also seen the development of selective BCL2, BCL(X)L and MCL1 inhibitors^19–21^, and the first entry of the selective BCL2 inhibitor, Venetoclax (ABT199) into the clinic. Since the selective targeting of anti-apoptotic BCL2 protein family members has become reality, we here provide proof-of-principle that DR_MOMP is capable of predicting responses of TNBC cells to genotoxic agents, and can be used as a stratification tool for the re-sensitization of resistant TNBC cells by BCL2 inhibitors.

## Results

### Characterization of cisplatin and paclitaxel responses in TNBC cell lines

**Fig. 1:**
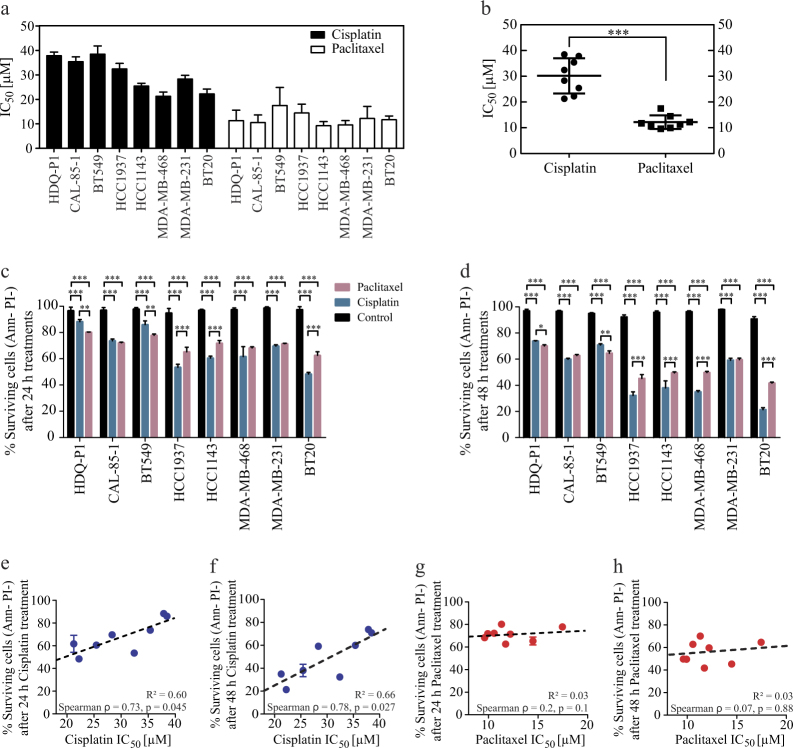
Cisplatin and paclitaxel sensitivity in a panel of TNBC cell lines Cells were incubated with increasing concentration of cisplatin or paclitaxel (from 0.3 to 1000 μM). After 24 h treatments MTT assay was performed and IC_50_ values calculated with GraphPad Prism using nonlinear regression with a variable slope fit function. (**a**) IC_50_ values for cisplatin (black) or paclitaxel (white) treatment. Means ± SD are shown for *n* = 3 experiments. (**b**) Same values were analyzed using Mann–Whitney test to show overall sensitivity of the drugs used (*** indicates a *p*-value < 0.001). (**c**,** d**) Percentages of surviving cells (Annexin V^−^/PI^−^ fraction) after 24 h and 48 h control, cisplatin (30 μM) or paclitaxel (10 μM) treatment analyzed through flow cytometry. Significance was assayed with a two-way ANOVA and Tukey post-test (* indicates a *p*-value < 0.05, ** indicates a *p*-value < 0.01, *** indicates a *p*-value < 0.001). Column represents mean ± SD for *n* = 3 experiments. **(e**,** f)** IC_50_ values for cisplatin were correlated to surviving cells levels after 24 h and 48 h cisplatin treatment, respectively, and analyzed through Spearman correlation test. **(g**,** h)** Same procedure was applied to paclitaxel treatment for 24 h and 48 h time points, respectively

We employed two clinically used genotoxic chemotherapeutics with different mechanisms of action, cisplatin and paclitaxel, to assess the power of DR_MOMP in predicting treatment responses in TNBC cells. We determined the sensitivity to both drugs in a panel of TNBC cell lines. Cells were incubated with increasing concentrations of the drugs, ranging from 0.3 to 1000 μM for 24 h, and cell viability was determined using an MTT assay (Supplementary Fig. S1). We observed a heterogeneous response to the treatments (Fig. 1a). In the presence of cisplatin, IC_50_ values ranged between 20 and 40 μM. HDQ-P1, CAL-85-1 and BT549 were less sensitive to the treatment (IC_50_ values between 35 and 40 μM) whereas BT20, MDA-MB-468 and HCC1143 showed a higher sensitivity, with IC_50_ values ranging between 20 and 26 μM (Fig. 1a). In the presence of paclitaxel, cell responses were less pronounced, with IC_50_ values ranging from 9 to 16 μM (Fig. 1a). Overall TNBC cell lines were more sensitive to paclitaxel compared to cisplatin after 24 h of treatment (Fig. 1b).

A single concentration for cisplatin or paclitaxel was selected to determine levels of cell death by flow cytometry. TNBC cells were treated with 30 μM cisplatin or 10 μM paclitaxel for 24 h and 48 h. Annexin V/PI staining was performed to indicate surviving, apoptotic and (secondary) necrotic cells. The concentrations used were selected based on the average IC_50_ values observed in Fig. 1b, to facilitate comparison and to approximate a clinical setup in which the patients will receive a standard dose of tolerated chemotherapy. After 24 h, cisplatin exerted a greater effect in HCC1937, HCC1143, MDA-MB-468, MDA-MB-231 and BT20 cells, decreasing cell survival between 40 to 50%. HDQ-P1, BT549 and CAL-85-1 cells were less affected. Responses to paclitaxel treatment were again less pronounced (Fig. 1c). In HDQ-P1 and BT549 cells, cisplatin was less effective compared to paclitaxel while in HCC1937, HCC1143 and BT20 cells cisplatin was more potent. No difference in cell survival was observed for CAL-85-1, MDA-MB-468 and MDA-MB-231 after treatment with cisplatin or paclitaxel (Fig. 1c). Similar results were observed for 48 h time point, except for cisplatin treatment in MDA-MB-468, which recorded significantly lower surviving cell levels when compared to paclitaxel (Fig. 1d).

We also investigated whether IC_50_ values correlated with cell survival when analyzed by Spearman’s correlation analysis. A correlation coefficient of 0.73 (*p*-value = 0.045, Fig. 1e) and 0.78 (*p*-value = 0.027, Fig. 1f) were observed for 24 and 48 h cisplatin treatment, respectively. No significant correlation was detected for paclitaxel (*p*-value = 0.1 and 0.8, Fig. 1g and h).

### Quantitative protein profiling shows heterogeneous BCL2 protein levels in TNBC cell lines

**Fig. 2:**
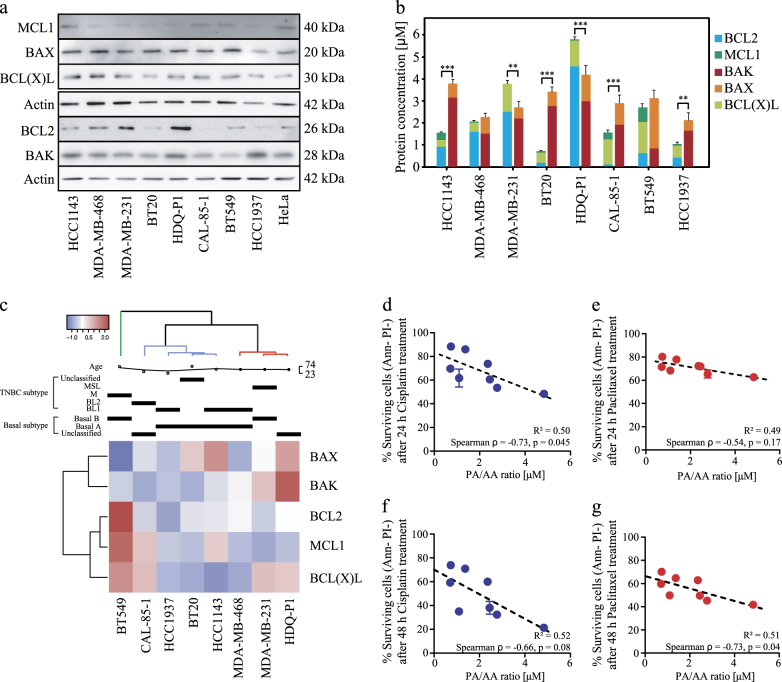
Quantitative BCL2 profiling of TNBC cell lines Cell lines were cultured and lysed in RIPA buffer. BCL2 profiling was performed comparing densitometric calibration curves from recombinant BCL2 proteins to densitometric signals of same proteins in HeLa cell lysates. (**a**) Western blots showing BCL2, BCL(X)L, MCL1, BAX, and BAK expression in TNBC cells. (**b**) Absolute concentrations of BCL2 proteins calculated relative to absolute BCL2 proteins concentration in HeLa. Bars represent means ± SD for *n* = 3 gels from 3 different lysates; two-way ANOVA with Bonferroni post-test was used to assess significance of pro-apoptotic against anti-apoptotic proteins (** indicates a *p*-value < 0.05, *** indicates a *p*-value < 0.001). (**c**) k-means clustering of BCL2 absolute protein concentrations. (**d**,** e**) Correlation between PA/AA ratio (pro-apoptotic to anti-apoptotic ratio calculated by dividing the sum of all pro-apoptotic proteins by the sum of anti-apoptotic proteins) and the percentage of surviving cells (Annexin V^-^/ PI^-^) after 24 h cisplatin and paclitaxel treatment, respectively. The correlation was tested using Spearman test. (**f**,** g**) Same analysis was performed for 48 h cisplatin and paclitaxel treatment, respectively

We next performed quantitative Western Blotting to determine the absolute protein levels for BCL2, BCL(X)L, MCL1, BAX and BAK in the cell lines (Table 1). Most cell lines exhibited higher, cumulative expression levels of BAK and BAX when compared to cumulative expression levels of anti-apoptotic BCL2 proteins, as previously observed in colon cancer cells^17^. An exception was HDQ-P1 and MDA-MB-231 cells where anti-apoptotic proteins dominated. BCL2 protein was detected at very low levels in CAL-85-1 cells, however this appeared compensated by high levels of BCL(X)L. In all cell lines except BT549 cells, BAK levels were higher than BAX levels. MCL1 exhibited by far the lowest expression level among all BCL2 proteins. HCC1143, MDA-MB-468, MDA-MB-231 and HDQ-P1 showed higher BCL2 levels compared to the other anti-apoptotic members, whereas the remaining cell lines showed higher BCL(X)L levels (Fig. 2a and b).

**Table 1:** Concentration values (in μM) for BCL2 proteins in the TNBC cells panel

	BCL2	MCL1	BAK	BAX	BCL(X)L
HCC1143	0.907	0.330	3.173	0.638	0.317
MDA-MB-468	1.609	0.051	1.518	0.761	0.416
MDA-MB-231	2.501	0.001	2.202	0.508	1.273
BT20	0.178	0.063	2.764	0.654	0.469
HDQ-P1	4.581	0.059	2.982	1.209	1.175
CAL-85-1	0.061	0.311	1.907	0.987	1.150
BT549	0.619	0.663	0.842	2.284	1.415
HCC1937	0.407	0.078	1.651	0.484	0.557

To better analyze expression similarities among the panel we clustered the different proteins using the complete linkage method. The cell lines were grouped in 3 different clusters; HDQ-P1, MDA-MB-231 and MDA-MB-468 showed similar expression patterns for BCL2, BCLXL, BAX and BAK proteins. HCC1143, BT20, HCC1937 and CAL-85-1 were instead grouped in a second cluster as the sum of pro-apoptotic proteins was significantly higher in this cluster than the sum of the anti-apoptotic members. BT549 cells were classified as the most different due to their higher levels of BAX, BCL(X)L and MCL1 compared to BAK and BCL2 (Fig. 2c).

### BCL2 protein levels poorly predict treatment responses

**Fig. 3:**
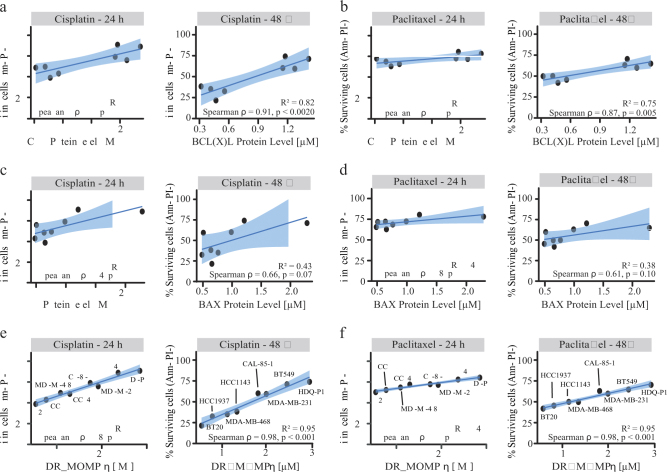
DR_MOMP is an excellent predictor of genotoxic chemotherapy responses (**a**,** b**) Correlation between BCL(X)L absolute protein levels from each cell line compared to % of surviving cells after 24 h and 48 h cisplatin and paclitaxel treatments, respectively, from flow cytometry data (Fig. 1c and d). (**c**,** d**) Same analysis performed for BAX protein levels. (**e**,** f**) Absolute proteins levels from BCL2 profiling were used as input to calculate DR_MOMP *η* score and correlated to % of surviving cells (Fig. 1c and d) after 24 h and 48 h cisplatin and paclitaxel treatments, respectively. All correlations were tested with Spearman test

BCL2 proteins have been described as important biomarkers for drug resistance and treatment responses^22^. Thus, the next step involved correlation analysis between BCL2 protein levels and treatment responses. We observed a positive significant correlation of BAX (Fig. 3c) after 24 and 48 h cisplatin treatment. Interestingly also BCL(X)L levels (Fig. 3a and b) correlated with 24 and 48 h cisplatin and paclitaxel treatment responses, respectively. Of note, we also observed a positive correlation between the sum of BCL2 and BCL(X)L protein levels and cisplatin/paclitaxel treatments (Supplementary Fig. S2a). No significant correlation was found with the remaining family members (Supplementary Fig. S2b, c and d).

The BCL2 expression levels across the panel also led to different pro-apoptotic to anti-apoptotic protein ratios (PA/AA ratio). Different BCL2 protein ratios have been previously used as a parameter to measure the sensitivity of a cancer cell population to undergo apoptosis^23–25^. We also tested whether this ratio could be used as a predictor for chemotherapy responses. We found that the PA/AA ratio negatively correlated with levels of surviving cells after 24 h cisplatin treatment (Fig. 2d, *R*^2^ = 0.50, *p*-value = 0.045) and 48 h paclitaxel treatment (Fig. 2g, *R*^2^ = 0.51, *p*-value = 0.04).

### DR_MOMP is superior in delivering accurately predictions of cell survival

We next investigated whether DR_MOMP, taking into account the signal network and protein interactions of BCL2 proteins, is more accurate in predicting responses of TNBC cells to cisplatin or paclitaxel. We observed an excellent, positive correlation between the DR_MOMP η score and response to 24 and 48 h cisplatin (Fig. 3e, *R*^2^ = 0.96 and 0.95, *p*-value < 0.001) or paclitaxel (Fig. 3f; *R*^2^ = 0.94 and 0.95, *p*-value = 0.001). Thus the model output outperformed both single protein levels and cumulative protein levels as predictors for cell survival, as it maintained the highest degree of correlation in all treatments.

### Selective BCL(X)L inhibition with WEHI-539 shows synergistic effects with cisplatin

**Fig. 4:**
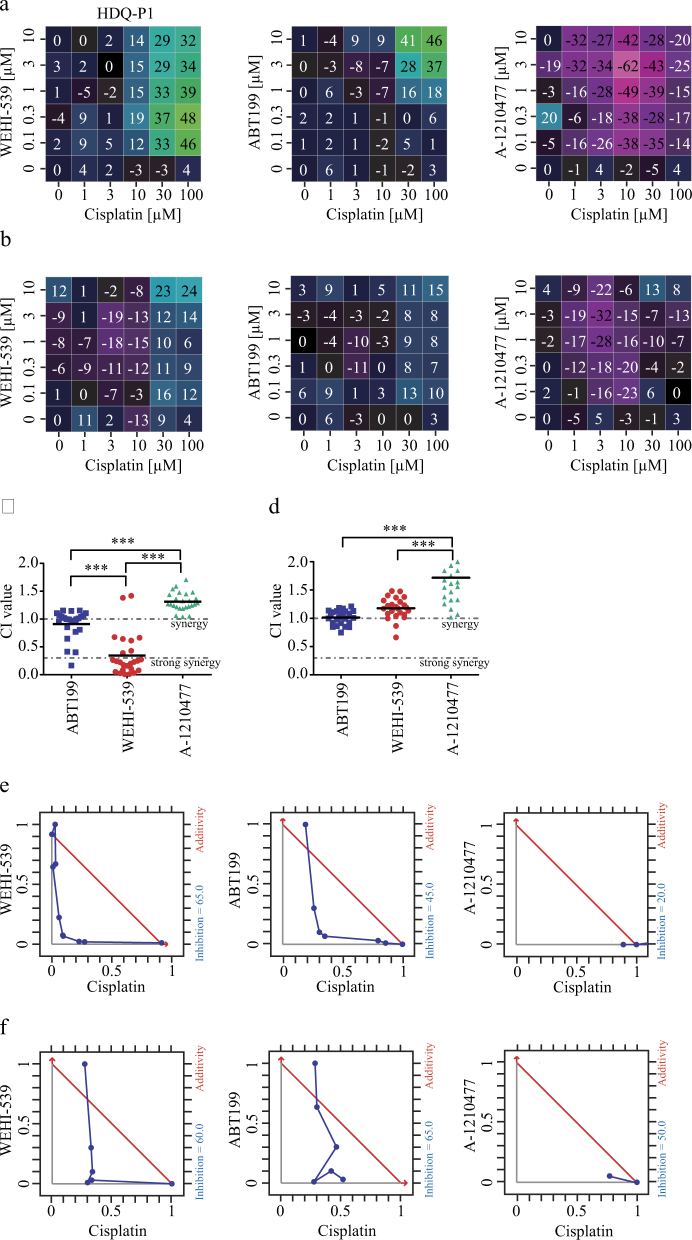
Synergistic effect of ABT199 and WEHI-539 in combination with cisplatin A 6 × 6 dose matrix assay was performed by treating HDQ-P1 and HCC1143 cells with increasing concentrations of cisplatin and BCL2 inhibitors. Cell viability was assessed after 24 h treatment with an acid phosphatase assay. Experiments were performed in triplicates (2 wells each repetition). (**a**,** b**) Heatmap of Loewe excess scores for WEHI-539, ABT199, and A-1210477 inhibitors in combination with cisplatin in HDQ-P1 and HCC1143 cells, respectively. Fraction affected % was calculated from viability and used to generate the scores. (**c**, **d**) Combination index values were calculated using Webb’s fractional product method for HDQ-P1 and HCC1143, respectively. A CI value lower than 1 means synergy while a CI lower than 0.3 is classified as strong synergy; CI values > 1 are considered as antagonistic. All experiments were performed in triplicate and results represent means ± SD. Values were analyzed using one-way ANOVA with Tukey post-test (*** indicates a *p*-value < 0.001). (**e**,** f**) Isobologram analysis for fraction affected % calculated from viability results for HDQ-P1 and HCC1143 cells, respectively

We next investigated whether ABT199, WEHI-539, and A-1210477, selective inhibitors of BCL2, BCL(X)L, and MCL1, respectively, were capable of enhancing responses of TNBC cell lines to genotoxic chemotherapy. We performed a 6 × 6 dose matrix assay to test for any synergistic activity between BCL2 inhibitors and cisplatin in the HDQ-P1 cell line which was among the most resistant to treatment with either cisplatin or paclitaxel (Fig. 1). After 24 h incubation with increasing concentrations of cisplatin in combination with increasing concentrations of each inhibitor, cell survival data (Supplementary Fig. S3) were analyzed using Loewe additivity analysis to evaluate drug interactions. As shown in Fig. 4a both WEHI-539 and ABT199 possessed synergistic activity when used in combination with cisplatin; in contrast A-1210477 showed no synergy at any concentration given. In the case of WEHI-539 high synergistic Loewe excess scores were observed for concentrations ranging from 0.1 to 10 μM in combination with 10–100 μM cisplatin. BCL2 selective antagonist showed synergistic Loewe excess scores at higher combination concentration, from 1 to 10 μM ABT199 with 30 or 100 μM cisplatin (Fig. 4a). We also repeated this assay for the HCC1143 cell line, which showed a higher response to cisplatin (Fig. 1a and c) and were placed in a different BCL2 cluster compared to HDQ-P1 (Fig. 2c). Again, we obtained similar results, except that high synergistic Loewe excess scores were observed for concentration ranging 0.1–10 μM in combination with 30–100 μM cisplatin for both WEHI-539 and ABT199.

We employed Webb’s fractional product method to calculate combination index (CI) values for combination treatments, and to confirm and to compare overall synergy among the drugs tested. CI value is an indicator of synergy (CI < 1), additivity (CI = 1) or antagonism (CI > 1). WEHI-539 showed the highest inhibition effect when compared to ABT199 and A-1210477 in HDQ-P1 cells (Fig. 4c). We observed that the selective BCL(X)L inhibitor possessed the lowest CI values when used in combination with cisplatin. ABT199 also acted synergistically with cisplatin but at a lower level. Interestingly, most CI values for MCL1 inhibitor fell into the antagonism category (Fig. 4c). No significant difference was observed when comparing CI values of ABT199 and WEHI-539 in HCC1143 cells (Fig. 4d). However, both inhibitors possessed lower CI values when compared to A-1210477. The isobologram analysis confirmed the synergistic effect between WEHI-539 or ABT199 in combination with cisplatin and highlighted that less WEHI-539 is needed to reach 50% inhibition compared to the other compounds. Again A-1210477 failed to show synergy in combination with cisplatin (Fig. 4e). Results were also confirmed for HCC1143 cell line; the isobologram analysis showed that WEHI-539 and ABT199 possessed similar synergistic effect in combination with cisplatin (Fig. 4f).

### DR_MOMP identifies the most effective BCL2 antagonist dose response for combination treatments

**Fig. 5:**
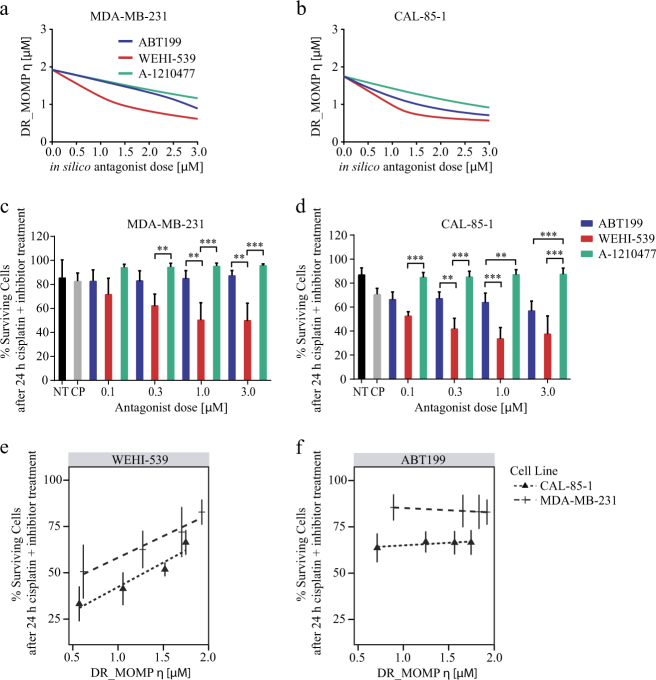
DR_MOMP predicts BCL2 inhibitors responses MDA-MB-231 and CAL-85-1 cell lines were treated with different concentrations of BCL2 inhibitors (0.1, 03, 1, and 3 μM) alone and in combination with 30 µM cisplatin. After 24 h treatments and staining with Hoechst and PI, the cells were imaged with a HCS platform and results analyzed with a CellProfiler pipeline to determine the levels of surviving cells. DR_MOMP was used to calculate the change in the predictive score assuming ABT199, WEHI-539, and A-1210477 binding kinetics. (**a**,** b**) DR_MOMP *η* score for ABT199, WEHI-539, and A-1210477 treatments in MDA-MB-231 and CAL-85-1 respectively. (**c**,** d**) HCS data for ABT199, WEHI-539, and A-1210477 in combination with 30 μM cisplatin for MDA-MB-231 and CAL-85-1 respectively. Two-way ANOVA with Tukey post-test was used to assess significance (** indicates a *p*-value < 0.01, *** indicates a *p*-value < 0.001). (**e**,** f**) Predicted DR_MOMP scores were interpolated by linear regression for the individual cell lines and plotted against % surviving cells after cisplatin and BCL2 antagonists treatment for WEHI-539 and ABT199 respectively. Correlation was analyzed with linear regression and Pearson’s coefficient

Finally we tested the ability of DR_MOMP to predict which selective BCL2 antagonist shows the highest synergistic activity with cisplatin, and to validate the in silico predictions by analysis of in vitro responses. We implemented the binding affinities of ABT199, WEHI-539 and A-1210477 to BCL2, BCL(X)L and MCL1 proteins into DR_MOMP based on published dissociation constants as described in Materials and Methods. Subsequently, we calculated the DR_MOMP score for MDA-MB-231 and CAL-85-1 after applying an in silico antagonist dose ranging between 0 and 3 μM (Fig. 5a) as these two cell lines possessed similar DR_MOMP *η* values and showed increased resistance to cisplatin treatments.

In silico predictions calculated that WEHI-539 substantially reduced the DR_MOMP score in both cell lines when genotoxic stress was applied. In contrast, CAL-85-1 cells were predicted to have a response to ABT199, which was not predicted in MDA-MB-231 cells (Fig. 5b). In both cell lines DR_MOMP predicted a poor response to MCL1 inhibition (Fig. 5a and b).

Our in silico predictions were validated by employing a high content analysis platform. We used Hoechst/PI double staining to quantify surviving cells after 24 h treatment with BCL2 inhibitors alone or in combination with cisplatin. As predicted in silico, WEHI-539 decreased cell survival when combined with 30 μM cisplatin, even at very low doses in MDA-MB-231 cells (Fig. 5c). No significant effect of ABT199 was observed. In contrast, both ABT199 and WEHI-539 reduced survival in CAL-85-1 cells, with no significant differences between the first and final treatments (0.1 and 3.0 μM concentrations). However, 0.3 and 1.0 μM treatments were found to be significantly different (Fig. 5d).

We further compared model predictions to in vitro data to determine whether DR_MOMP is able to calculate maximal response regions. We found a linear correlation between % of surviving cells and the DR_MOMP scores for WEHI-539 in both cell lines (Fig. 5e), suggesting that we can employ DR_MOMP to predict a dose response to BCL(X)L inhibition. The Pearson’s correlation coefficients were 0.96 and 0.98 for CAL-85-1 and MDA-MB-231, respectively. Also in other resistant TNBC cell lines investigated, the change in DR_MOMP score in response to WEHI-539 was equivalent to the change in survival in vitro (Supplementary Fig. S2a). We also found a linear correlation between cell survival and DR_MOMP scores for ABT199 combined with cisplatin in the responsive CAL-85-1 cells (Pearson’s correlation coefficients: 0.84) (Fig. 5f). MCL1 was not included in the analysis as no differences in cell survival were observed. Moreover, we correlated BCL2 protein levels to combination treatment responses and found no significant correlation in most of the cases. Of note, we observed that BCL(X)L protein levels positively correlated with surviving cell levels after ABT199 plus cisplatin treatment and PA/AA ratio negatively correlated with WEHI-539 and cisplatin treatment (Supplementary Fig. S4 d and h). Finally, we also tested for synergy interaction in DR_MOMP, between the in silico BCL2 inhibitors and genotoxic stress. We calculated the amount of pores formed when applying increasing concentrations of BCL2, BCL(X)L and MCL1 selective inhibitors in combination with increasing dose of genotoxic stress. As shown in Supplementary Fig. S5a an increased amount of pores is formed at lower concentration of combined WEHI-539 and genotoxic stress, when compared to ABT199 and A-1210477. We also calculated the CI values to account for synergy and again found that higher synergistic interactions (lower CI values) are observed at lower concentration of WEHI-539 in combination with genotoxic stress, when compared to ABT199 and A-1210477 (Supplementary Fig. S5b).

## Discussion

Despite recent advances, TNBC treatment still represents a major challenge and few biomarkers have been studied to stratify patient responses. Previous studies on the effect of BCL2 proteins in breast cancer largely focused on individual members of the BCL2 network^13,16,26^. Our study demonstrates that a systems approach can be successfully used to predict TNBC cellular responses to genotoxic chemotherapy in vitro and their synergy with selective BCL2 inhibitors.

TNBC cells were found to show heterogeneity in treatment responses; IC_50_ values for cisplatin highlighted a good correlation with treatments responses. However, paclitaxel IC_50_, failed to show any predictive value. Although IC_50_ calculations are widely used to test a set of treatments they are highly variable and rely on factors such as incubation time, drug concentration, and the technique employed.

As in colon cancer, also TNBC cells display overexpression of the BCL2 proteins at different rates;^17,18^ tumors often present BCL2 gene amplification and this relates itself to increased protein levels^27^.

Despite MCL1 being identified as an important survival factor in TNBC^26^, our quantitative study found that BCL(X)L was a better prognostic factor for treatment responses. Indeed it was recently shown that BCL(X)L is more potent in protecting the cells against genotoxic stress when compared to BCL2 or MCL1^28^. Interestingly, we also observed a correlation between BAX and treatment responses which would contradict the hypothesis that higher pro-apoptotic levels will result in increased cell death after treatment. We found no significant correlation between BCL(X)L and BAX protein levels, excluding the possibility that these two proteins are transcriptionally co-regulated, however BAX protein levels may correlate with other factors that determine responses to cytotoxic agents. BCL(X)L has also been implied in BAX retro-translocation from the mitochondria to the cytosol in order to regulate mitochondrial priming^29,30^ highlighting a sub-cellular shuttling mechanism that could explain why these proteins are better predictor for treatment outcome. All other BCL2 proteins poorly predicted chemotherapeutic sensitivity even when the ratio between pro and anti-apoptotic was considered in the analysis.

In contrast, DR_MOMP successfully predicted cell survival as the stress dose highly correlated with treatment responses in TNBC cell lines. The advantage of our system biology approaches over other biomarkers is the possibility to integrate expression levels with network information and biochemical data such as binding affinities and production/degradation rates. Indeed, DR_MOMP outperformed both single protein and combinatorial biomarkers.

Several drugs that target BCL2 proteins have been developed during recent years. ABT737 was one of the first agents to be developed with high affinity towards BCL2, BCL(X)L, and BCLW, however this compound showed a low solubility and poor oral availability. An oral active version, ABT263/Navitoclax, was subsequently developed, but its clinical utility is restricted due to the increased risk of thrombocytopenia as a consequence of its BCL(X)L inhibiting activity^31^, an important protein for platelet survival. Previous studies showed the efficacy of a BCL(X)L selective inhibitor in combination with genotoxic chemotherapy to induce cell death in solid tumors such as osteosarcoma, ovarian, colon, and breast cancer^32–35^. Our findings revealed that BCL(X)L inhibition was very potent in decreasing cell viability in most TNBC cells when used together with cisplatin. This approach could be successfully used to re-sensitize cancer cells to cell death in patient resistant to chemotherapy. Of note, our study demonstrates that DR_MOMP is able to calculate, case specifically the most efficient dose and best dynamic range that reduces the viability of TNBC cells. This raises hope that pharmacodynamics stratification tool such as DR_MOMP may not only identify patients responding to BCL(X)L inhibition, but may also be able to define those that may benefit from low concentrations of BCL(X)L selective inhibitors, thereby reducing unwanted side effects such as thrombocytopenia.

Recently the selective BCL2 inhibitor ABT199 was approved for the treatment of chronic lymphocytic leukemia with 17p deletion^36^. This drug is specific for BCL2 and has no impact on platelets survival. To date ABT199 is used in different clinical trials alone and in combination with alkylating agents or CD20-targeted antibodies^31^. Although ABT199 is often considered to be effective only in BCL2 dependent cancers, our study indicates that it can also be used to increase cisplatin effectiveness in TNBC cells that express both BCL(X)L and BCL2, albeit at higher concentration than WEHI-539. Treatment with ABT199 may nevertheless be a viable option, as BCL2 inhibition may induce less side effects. Thus systems approaches such as DR_MOMP are needed to address which patient will respond to BCL2 inhibition when co-expressing BCL(X)L.

On a cautionary note, it should also be considered that BCL-2 antagonists may exert additional effects unrelated to apoptosis initiation. It was previously shown that BCL2, MCL1, and BCL(X)L regulate different aspects of cellular metabolism^37–40^, mitochondrial energetics, fusion/fission, and morphology, thereby potentially limiting the effects of the model. Another limitation is that the model does not yet incorporate pharmacokinetics. Inclusion of such parameters will further enhance the clinical applicability of DR_MOMP, which will require validation in preclinical models such as patient-derived xenografts.

In conclusion, we here demonstrate that our approach can be used to predict cellular responses to chemotherapy in TNBC and lay down the foundation for the deployment of DR_MOMP as a pharmacodynamics stratification tool for BCL2 antagonist.

## Materials and methods

### Materials and reagents

Fetal bovine serum, RPMI 1640 medium, insulin, p-Nitrophenyl Phosphate Substrate (pNPP), Thiazolyl Blue Tetrazolium Bromide (MTT), dimethyl sulfoxide (DMSO), Hoechst 33588, and propidium iodide (PI) came from Sigma-Aldrich (Dublin, Ireland). DMEM medium was purchased from Lonza (Analab Ltd, Lisburn, United Kindom) and DMEM/F12 from Gibco (Biosciences, Dún Laoghaire, Ireland). ABT199 was purchased from Active Biochem (Maplewood, NJ, USA), WEHI-539 from ChemScene (South Brunswick, NJ, USA) and A-1210477 was obtained from AbbVie (North Chicago, IL, USA). Cisplatin and paclitaxel were purchased from Selleckchem (Stratech Scientific Ltd, Newmarket, United Kindom).

### Cell lines

HCC1937, HCC1143, MDA-MB-231, and MDA-MB-468 were cultured in RPMI-1640 supplemented with 10% FBS, 1% L-Glutamine and 1% penicilin/streptomycin; same medium with the addition of 0.023 UI Insulin and 10 mM HEPES was used for BT549 cell line. DMEM supplemented with 10% FBS, 1% L-Glutamine, and 1% penicilin/streptomycin was used for HDQ-P1, HeLa, and CAL-85-1 (with the addition of 1 mM pyruvate). BT20 were grown in DMEM/F12 supplemented with 10% FBS, 1% L-glutamine and 1% penicilin/streptomycin. All cell lines were incubated at 37 °C in humidified atmosphere with 5% of CO_2_. Cell lines were authenticated by STR typing from Source Bioscience (Nottingham, United Kindom).

### MTT assay

The MTT assay was used to determine cisplatin and paclitaxel IC_50_ values. TNBC cell lines were seeded at a density of 3×10^4^ cells for well on 96-well plates at 37 °C and treated with increasing concentration of cisplatin and paclitaxel (from 0.3 to 1000 µM). After 24 h 20 μL of 5 mg/mL MTT (in 1X PBS) was added to each well and the plate incubated at 37 °C for 4 h. Consequently medium was removed and crystals were suspended in 100 μL DMSO. Absorbance at 570 nm was recorded on a Multiskan® EX plate reader (Thermo Scientific, Dublin, Ireland). IC_50_ values were calculated with Prism (GraphPad, La Jolla, CA) by using a nonlinear regression with a variable slope fit function.

### Synergy calculations

Acid phosphatase assay was used to measure cell viability based on the conversion of pNPP to p-nitrophenol by cytosolic acid phosphatase^41^. Cells were grown in a 96 well plate at a density of 1.5×10^4^ cells per well and treated with increasing concentrations of cisplatin in combination with increasing concentrations of ABT199, WEHI-549, or A-1210477. After 24 h treatment medium was removed and each well was washed once with 200 μL of 1X PBS. To each well, 100 μL of assay buffer (0.1 M sodium acetate at pH 5.0, 0.1% Triton X-100, and 7.25 mM p-nitrophenyl phosphate) was added. The plates were then incubated at 37 °C for 2 h. The reaction was finally stopped with the addition of 50 μL and color development was assayed at 405 nm using a Multiskan® EX plate reader. The non-enzymatic hydrolysis of the pNPP substrate was also determined by including wells with the assay buffer and without any cells. An excel template was used to calculate the fraction affected from viability percentages and the results were analyzed with the web version of Chalice Analyzer (Horizon Discovery) to calculate Loewe model matrix and isobologram^42^. Combination index values were calculated using Webb’s fractional product method^43^.

### Western blotting and BCL2 profiling

Cells were seeded at a density of 1×10^6^ and let to attach. After 24 h, cells were scraped, collected, and lysed in RIPA buffer (150 mM NaCl, 1.0% IGEPAL® CA-630, 0.5% sodium deoxycholate, 0.1% SDS, 50 mM Tris, pH 8.0, protease and, phosphatase inhibitors mix 1:100) to obtain whole cell lysates. Protein concentration was determined with micro BCA (bicinchoninic acid) assay (Pierce) and a total of 30 µg of protein loaded into a SDS-gel after complete denaturation at 90 °C for 10 min in Laemmli buffer. The samples were then transferred to nitrocellulose membrane and blocked in 5% milk in TBS-T for 1 h. Primary antibodies to MCL1 (1:1000; BD Biosciences), BCL2 (1:100; Santa Cruz Biotechnology), BCL(X)L (1:250; Santa Cruz Biotechnology), and actin (1:5000; Sigma Aldrich) were mouse monoclonal. Antibodies to BAK (1:250; Santa Cruz Biotechnology) and BAX (1:1000; Upstate Biotechnology) were rabbit polyclonal. The horseradish peroxidase (HRP)-conjugated secondary antibodies were from Jackson ImmunoResearch (1:5000). Detection of protein bands was carried out using chemiluminescence (EMD Millipore) on a LAS-3000 Imager (FUJIFILM UK Ltd. System). BCL2 profiling and absolute protein concentration was carried on as previously described through quantitative western blotting^17^. Briefly, standard curves were constructed with varying concentrations (0.1–10.0 ng) of recombinant BCL2 proteins, and varying concentrations of HeLa extract. From these western blots, calibration curves for each protein were established, plotting blot intensity to mass of loading. Cellular concentrations for BCL2 proteins in TNBC cellular lysates were calculated from calibration curves, considering HeLa cell volume and the appropriate molecular weights for BAK, BAX, BCL2, BCL(X)L, and MCL1. We assumed a HeLa cell volume of 3.1 pL from previous imaging study^44^. Densitometry was conducted using ImageJ 1.45 s (National Institutes of Health, Bethesda, MD, USA)

### Flow cytometry

Cells were seeded on a 24 well plate at a density of 6×10^4^ cells for well and treated with 30 µM cisplatin and 10 µM paclitaxel for 24 h and 48 h at 37 °C. After incubation time cells were collected by tripsinization and stained with Annexin V^−^FITC and PI (Biovision) for 20 min at room temperature in dark condition and analyzed using a CyFlow ML (Partec) flow cytometer and FloMax software. A minimum of 10,000 events were recorded for each sample. Surviving cells were defined as the fraction of Annexin V and PI negative cells. The percentage of apoptotic cells was defined as Annexin V positive/ PI negative plus Annexin V positive/ PI positive.

### High content screening microscopy

Cells were seeded in a Nunc Micro Well 96 well optical bottom plate (Thermo Scientific) at a density of 1.5×10^4^ cells per well. The day of the treatment cells were incubated in medium with 1 μg/mL Hoechst 33588 and 1 μg/mL PI. After 24 h treatment, plates were imaged at 30 fields of view per well using a Cellomics Arrayscan VTI (Thermo Scientific) microscope set up with a temperature of 37 °C and 5% of CO_2_ in humidified atmosphere. Images were taken at a resolution of 0.645 μm/pixel using a ×10 PlanApo objective lens (NA 0.45), a 120 W Hg arc illumination source with 12% ND filter (EXFO, Chandlers Ford, UK) and a monochrome CCD camera (Orca-AG, Hamamatsu Photonics, Hertfordshire, UK). The following filters sets were used: Hoechst excitation 387 ± 11 nm, emission 447 ± 30 nm; PI excitation 560 ± 12 nm, emission 620 ± 60 nm all using a HC-Quad band beam splitter with transition wavelength of 410, 504, 582, and 669 nm (Semrock, AHF, Germany). Images were analyzed using a customized processing pipeline to identify nuclei with Hoechst staining (total cell number) and nuclei of dead cells (PI positive) using CellProfiler r2.2.0^45^.

### DR_MOMP

DR_MOMP is an ordinary differential equation (ODE)-based systems model of BCL-2 protein interactions comprised of 126 reactions and 71 protein species that delivers a numeric score indicative of the stress dose required to induce MOMP in individual cancer cells^17^. BCL2 profiles of BT20, BT549, CAL-85-1, HCC1937, HCC1143, MDA-MB-231, MDA-MB-468, and HDQ-P1 cells were used as input to determine the DR_MOMP score. Production rates of BIM, PUMA, and NOXA proteins were assumed to be identical and modeled by a step function at a constant rate for 12 h. The entire protein production during this period was defined as (protein) dose *η* and compared to cell death rates in TNBC cells. A full description of DR_MOMP is given in the supplemental material of Lindner et al. 2013^17^ and in the supplementary method section of this study. Binding kinetics (k_forward_) of the selective BCL2 antagonists ABT-199/Venetoclax (BCL2), WEHI^−^539 [BCL(X)L], and A-1210477 (MCL1) were implemented in DR_MOMP as previously described for ABT737 and Apogossypolone (ApoG2)^17^. Dissociation constants (*K*_D_) used to determine binding kinetics were originally described in the literature^19–21^. To predict the impact of ABT199, WEHI-539, and A-1210477 on the tumor cells’ sensitivity to genotoxic stimuli, DR_MOMP scores were determined with different doses of the antagonists, ranging from 0 to 6 μM, and corrected by the factor 0.5 to account for drug degradation and active drug extrusion. The antagonist doses were applied in silico at the same time and for the same duration as the genotoxic stress (12 h)^17^.

### Statistical analysis

Data are given as means ± S.D. (standard deviation). Correlations were assessed using Spearman’s rank correlation analysis. For statistical comparison, two-way analysis of variance (ANOVA) or one-way analysis followed by Tukey’s *post hoc* test were employed. *p*-values < 0.05 were considered to be statistically significant.

## Electronic supplementary material

Supplementary figures

DR_MOMP modeling supplement
