# Supplementary material for: Systems modeling accurately predicts responses to genotoxic agents and their synergism with BCL-2 inhibitors in triple negative breast cancer cells
Source: Cell Death Dis. 2018 Jan 19;9(2):42. doi: 10.1038/s41419-017-0039-y (PMC5833806; doi:10.1038/s41419-017-0039-y)

Supplementary figures to

Systems modelling accurately predicts responses to genotoxic agents and their synergism with BCL-2 inhibitors in triple negative breast cancer cells

Federico Lucantoni[1,2], Andreas U. Lindner[1,2], Norma O'Donovan[3], Heiko Düssmann[1,2], and Jochen H.M. Prehn[1,2].

[1]Department of Physiology & Medical Physics, Royal College of Surgeons in Ireland, Dublin 2, Ireland.
[2]Centre for System Medicine, Royal College of Surgeons in Ireland, Dublin 2, Ireland.
[3]National Institute for Cellular Biotechnology, Dublin City University, Dublin 9, Ireland.

Supplementary Figure Legends

Supplementary Figure S1. Dose-Response curves for chemotherapeutic treatments. (a and b) MTT dose-response curves for TNBC cell lines after 24 hours cisplatin and paclitaxel treatments respectively, used to calculate the IC_{50} values.

Supplementary Figure S2. BAK, BCL2 and MCL1 poorly predict cell survival after treatments. (a, b, c and d) Correlation between BCL2+BCL(X)L, BAK, BCL2 and MCL1 absolute protein levels from each cell line compared to % of surviving cells after cisplatin and paclitaxel treatments, respectively, from flow cytometry data (Figure 1c). All correlations were tested with Spearman test.

Supplementary Figure S3. Dose matrix assay for cisplatin in combination with BCL2 proteins inhibitors. (a and b) Fraction affected % after treatment of HDQ-P1 and HCC1143 cells, respectively, with increasing concentrations of BCL2 inhibitors in combination with increasing concentrations of cisplatin. This data was used to calculate Loewe excess scores in Figure 4.

Supplementary Figure S4. DR_MOMP predictions for combination treatments. (a and b) Percentages of surviving cells related to DR_MOMP η values after combination of cisplatin with WEHI-539 and ABT199, respectively, in BT549, CAL-85-1, HDQ-P1 and MDA-MB-231. **(c, d, e, f, g, h, i)** Correlation between surviving cells after combination treatments (ABT199, WEHI-539 and A-1210477 in combination with cisplatin) and BCL2, BCL(X)L, MCL1, BAX, BAK, PA/PP ratio and BCL2+BCL(X)L proteins levels, respectively.

Supplementary Figure S5. DR_MOMP predicts synergy for combination treatments in HDQ-P1 cells. (a) The amount of pore induced by increasing *in silico* concentration of WEHI-539, ABT199 or A-1210477 in combination with increasing genotoxic stress was calculated in DR_MOMP. **(b)** The level of pores was then analysed with the Webb's fractional product method to obtain CI values and test for synergy.

Supplementary Figures

Supplementary Figure S1

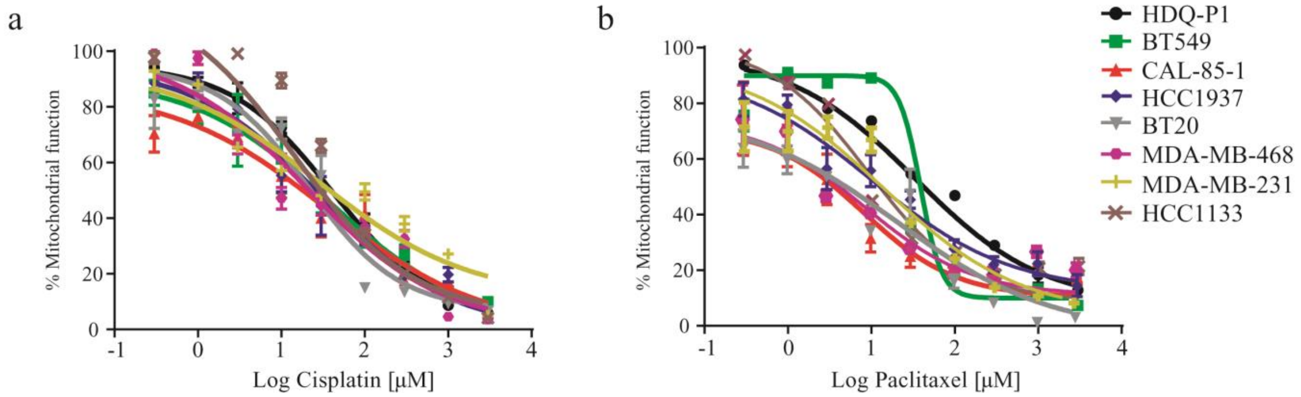

Supplementary Figure S2

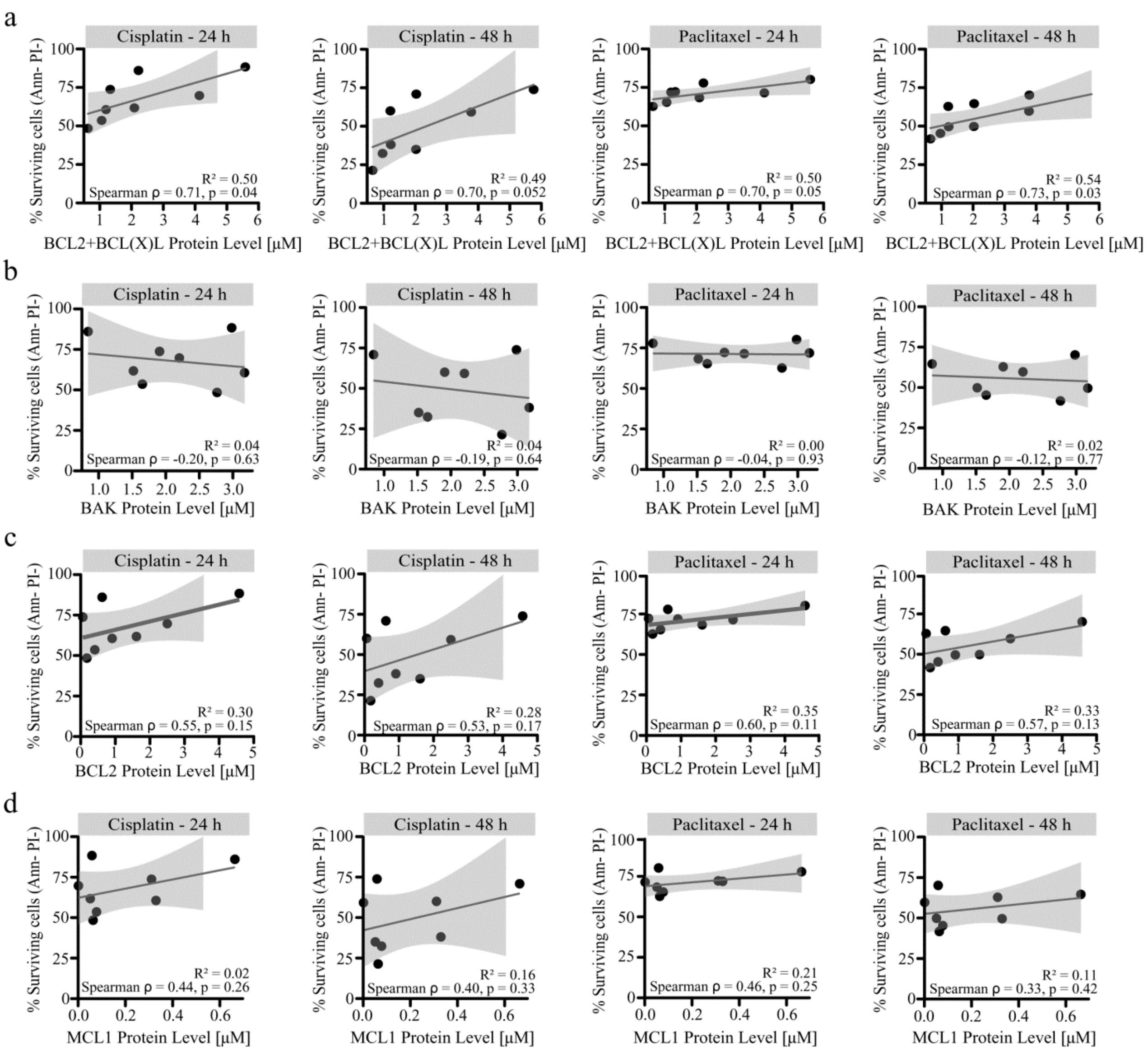

Supplementary Figure S3

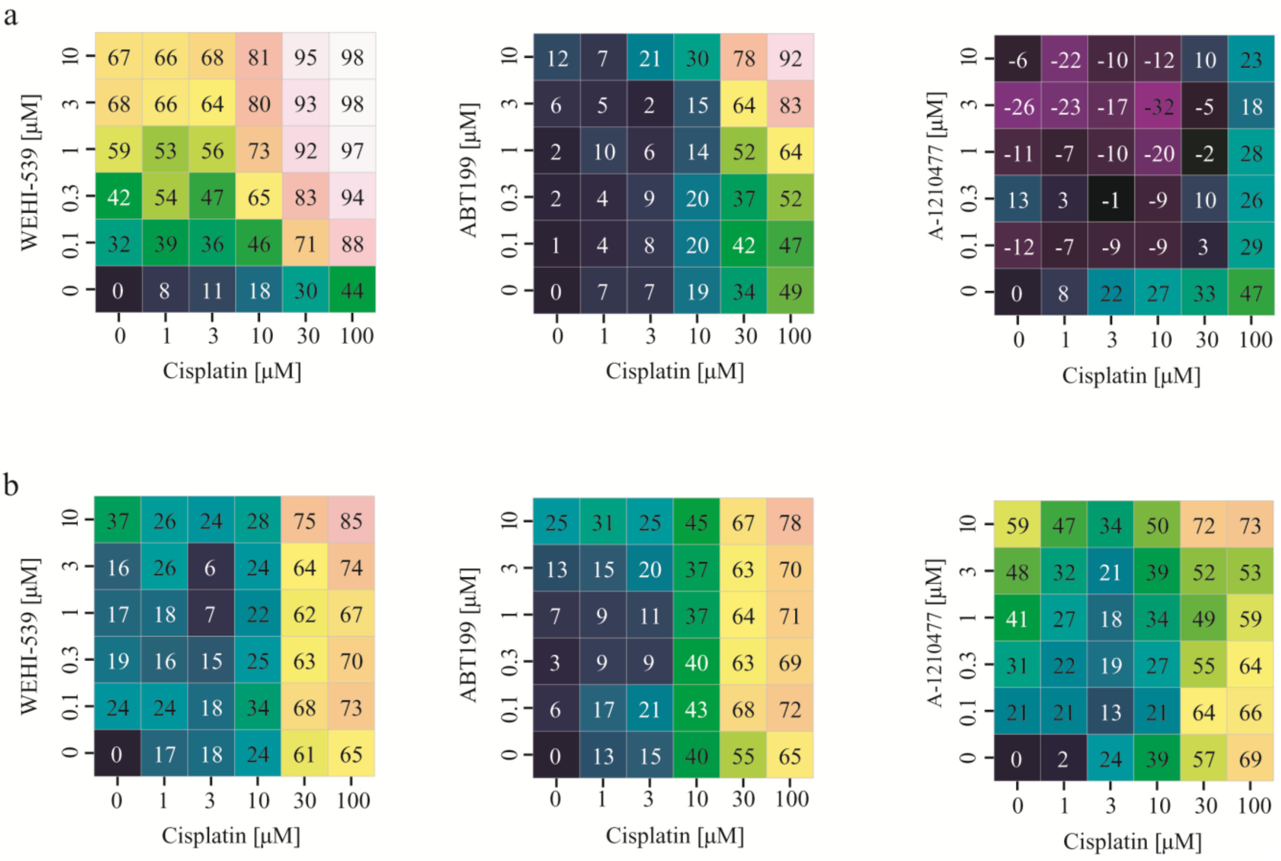

Supplementary Figure S4

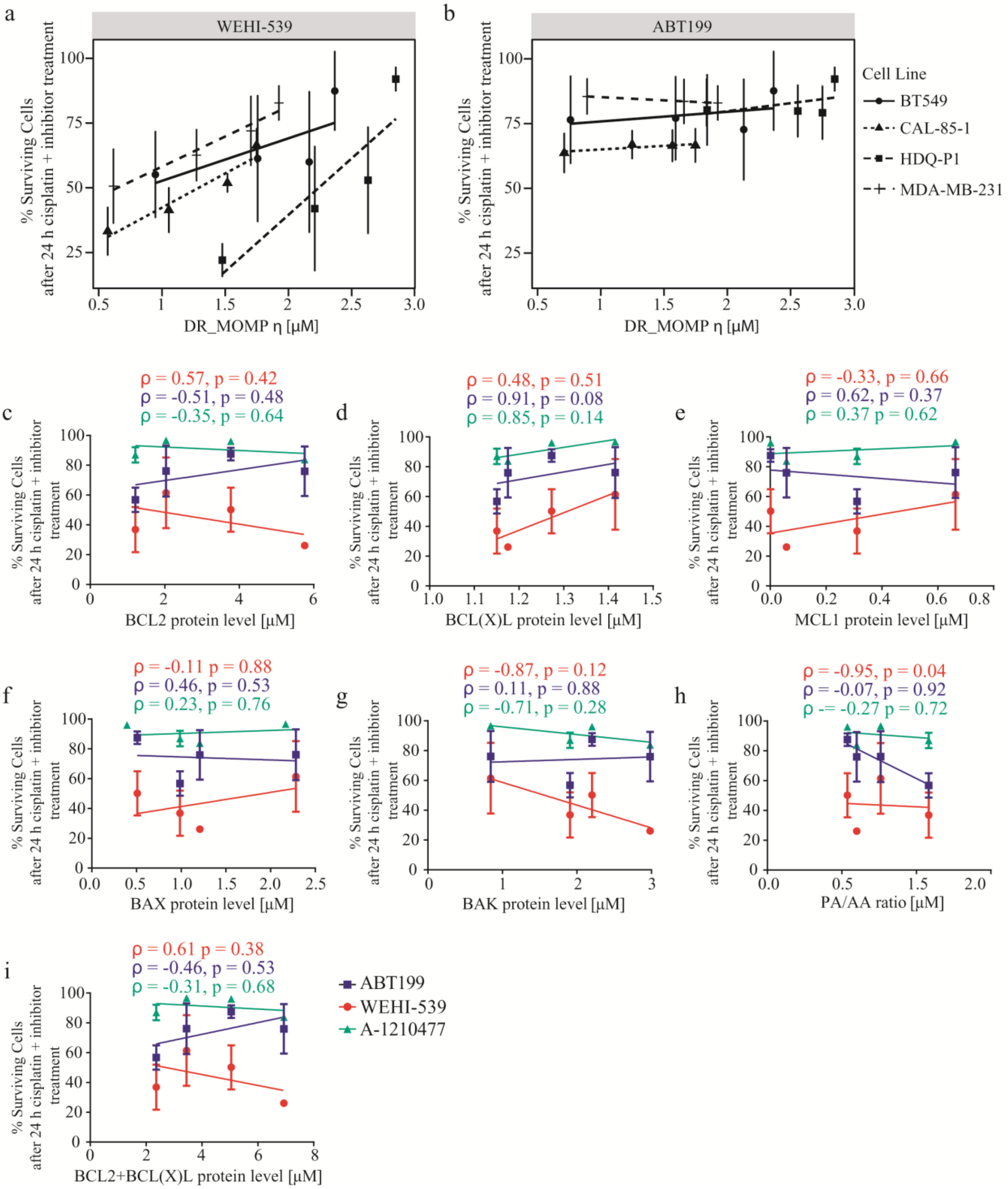

Supplementary Figure S5

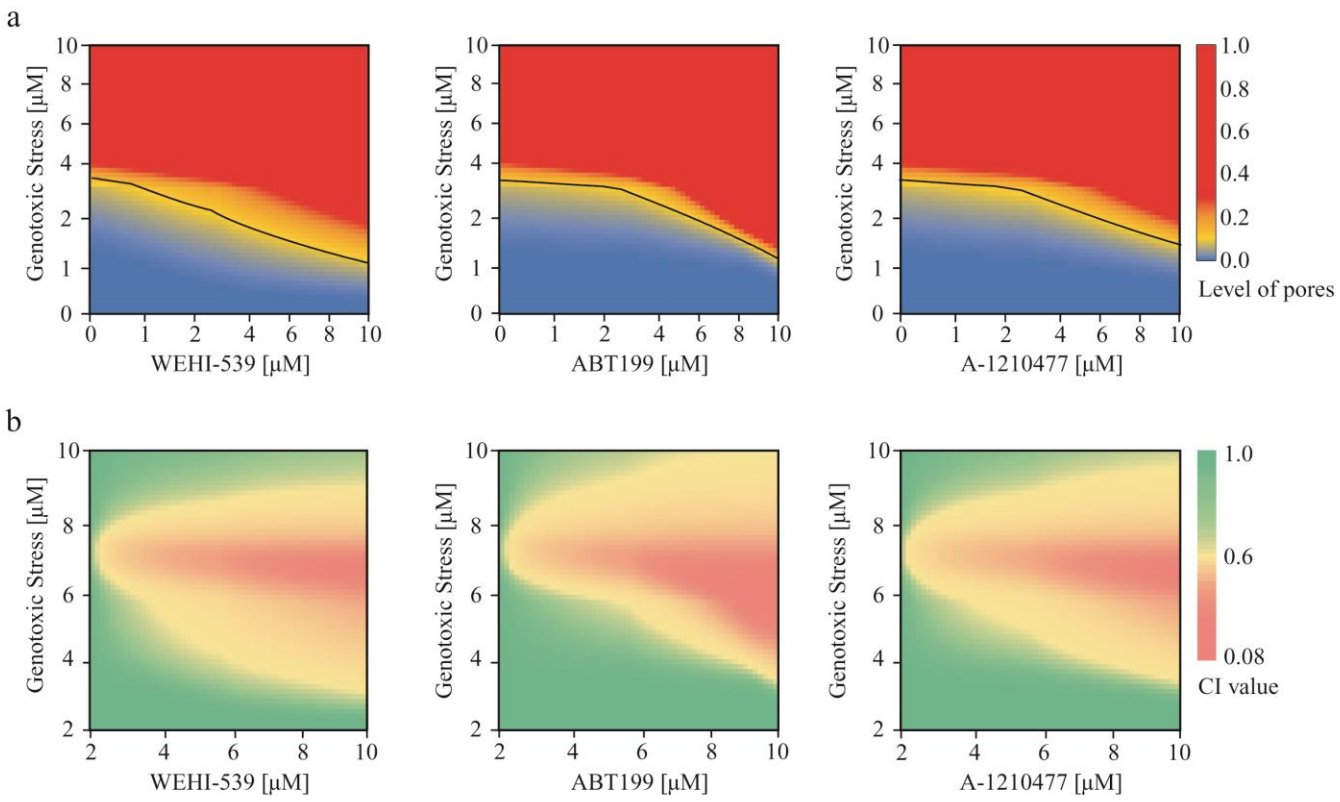

Supplement: Supplementary file 1 — Supplementary figures [file 41419_2017_39_MOESM1_ESM.pdf]
