# Supplementary material for: Systems modeling accurately predicts responses to genotoxic agents and their synergism with BCL-2 inhibitors in triple negative breast cancer cells
Source: Cell Death Dis. 2018 Jan 19;9(2):42. doi: 10.1038/s41419-017-0039-y (PMC5833806; doi:10.1038/s41419-017-0039-y)
Supplement: Supplementary file 2 — DR_MOMP modeling supplement [file 41419_2017_39_MOESM2_ESM.pdf]

Supplementary methods to

Systems modelling accurately predicts responses to genotoxic agents and their synergism with BCL-2 inhibitors in triple negative breast cancer cells

Federico Lucantoni[1,2], Andreas U. Lindner[1,2], Norma O'Donovan[3], Heiko Düssmann[1,2], and Jochen H.M. Prehn[1,2].

[1]Department of Physiology & Medical Physics, Royal College of Surgeons in Ireland, Dublin 2, Ireland.

[2]Centre for System Medicine, Royal College of Surgeons in Ireland, Dublin 2, Ireland.

[3]National Institute for Cellular Biotechnology, Dublin City University, Dublin 9, Ireland.

Supplementary Modelling Details

Mathematical modeling overview

This supplementary modelling overview was used from Lindner *et al* (1) and adapted to include the new BCL2 inhibitors section. The signaling pathway of the BCL2 family proteins was modeled by a pseudo-reaction network. Using mass-action kinetics, this network was subsequently translated into a set of ordinary differential equation (ODEs) which describe the changes in concentration of a single protein or a single protein complex at a given time point t (denoted as 'reaction rate', or 'rate', hence further). ODEs were parameterized using cell specific concentrations and kinetic constants as input. Subsequently, ODEs were solved using MATLAB 7.3 (MathWorks, USA, R2007b, 7.5.0.342) and its function *ode15s*.

We translated the modeled protein-protein interaction from a general reaction notation to ODEs by a detailed guideline listed in Supplementary Table 1. The guideline is grouped into different reaction classes. In a nutshell, the degradation rate of a protein was calculated by multiplying the actual protein concentration by the degradation constant k_{deg} and this rate was subtracted from the actual protein concentration (Supplementary Table 1a). Protein turnover was realized by adding a production k_{prod} rate to the degradation rate (Supplementary Table 1b). Proteins that sustain turnover were modeled to reach equilibrium concentrations that were given by the interplay of protein production and protein degradation. As a consequence the equilibrium that was attained was given by the ratio of production rate and degradation constant (k_{prod} / k_{deg}). Protein translocation was modeled by multiplying the actual protein concentration by the kinetic constant k (Supplementary Table 1c). The change in concentration due to a protein-protein interaction, such as binding, was modeled by multiplying the product of the concentration of all interacting proteins by the reaction specific constant k (Supplementary Table 1d). This was irreversible unless a backward equation was modeled with a separate reaction specific constant (Supplementary Table 1e). The ratio of the backward constant $k_{backward}$ and the first / forward constant $k_{forward}$ is defined as dissociation constant K_D. A Low value of K_D indicates a high affinity of the interacting proteins. *Vice versa* a high value indicates a low affinity.

Modeling of BCL2 protein reactions

Proteins of the BCL2 protein family were modeled to be located and to interact at the mitochondrial outer membrane (2, 3). As only exception cytosolic BAX was modeled to have a cytosolic fraction BAX_c (4).

As the first type of protein interaction, protein degradation was modeled for all proteins and protein complexes except for BAK, BAX and VDAC2 and their complexes (Supplementary Table 2). Degradation constants k_{deg} were calculated with the equation $k_{deg} = \ln(2)/60*t_{1/2}$ from half-life times $t_{1/2}$. We obtained the protein half-life time of the proteins BCL2, BIM, MCL1, NOXA, PUMA and tBID from literature (5-11). We considered for BCL(X)L the same half-life time as for BCL2 (300 minutes) due to the similar amino acid sequence of BCL2 and BCL(X)L (EMBOSS Needle alignment, similarity 53%). Because MCL1 is subject to rapid degradation (5, 7), the degradation constant for MCL1 was modeled to drop from 45 minutes to 17 minutes upon induction of BIM, PUMA and NOXA stress at time point t = 0h after stress induction. We modeled stable levels of VDAC2 (12) as well as for BAK and BAX (protein half-life time > 22 hours (12, 13)) for model simplification. Degradation rate of BCL2 hetero-dimers MCL1~BIM, BCL2~tBID, BCL(X)L~tBID, MCL1~PUMA and MCL1~NOXA were obtained from literature (2-5). We modeled the same half-life time for the hetero-dimers of anti-apoptotic BCL2 and BCL(X)L with BIM, NOXA and PUMA, and MCL1~tBID as for BCL2 and BCL(X)L with tBID.

We modeled reversible bindings between the anti-apoptotic proteins BCL2, BCL(X)L and MCL1, and the pro-apoptotic proteins BAK, BAX, BIM, tBID, PUMA and NOXA (Supplementary Table 3). For the binding of BIM to BCL2, BCL(X)L, and MCL1, the dissociation constants K_D (K_D is defined as $k_{backward}$ / $k_{forward}$) as well as the constants $k_{forward}$ and $k_{backward}$ were taken from (14). For the binding of PUMA, NOXA and tBID to BCL2, BCL(X)L, and MCL1, K_D values were taken from literature (4, 15-21). The backward kinetic constants $k_{backward}$ for the reversible binding of BCL2, BCL(X)L, and MCL1 with each BAK, BAX,

NOXA, PUMA and tBID were modeled to be the same as those for BIM. The forward dissociation constant was calculated by $k_{forward} = k_{backward} / K_D$.

Inactive BAX_c was modeled to be only present in the cytosol. Hence, no interaction of inactive BAX_c with anti-apoptotic BCL2 family proteins (22) was modeled. BAX_c activation by BH3-only proteins BIM, PUMA (and tBID) was modeled in a two-step procedure. Therefore BIM, PUMA (and tBID) were first modeled to reversibly bind with an fitted dissociation constant K_D of 100 nM that is consistence with Dai *et al*. (23) and forward kinetic constant $k_{forward}$ of $2.57x10^{-6}$ $nM^{-1}s^{-1}$. The backward constant was calculated by $k_{backward} = K_D * k_{forward}$. Formed activator~$BAX_c$ hetero-dimers were modeled to irreversibly activate BAX_c* followed by an instant release of BAX_c* with an estimated half-life time of 1/10 minute (Supplementary Table 4a and b). Upon its activation, BAX_c* was modeled to translocate into the mitochondrial outer membrane (MOM) with a fast single cell kinetic that was determined by our lab previously (24) (Supplementary Table 4c).

In the model, BAK activation was modeled with the same reactions and kinetics (Supplementary Table 4a and b) as BAX. However, these interactions were considered to take place at the mitochondrial membrane and therefore no translocation was modeled (25). Unlike BAX, BAK was modeled to interact with the Voltage-dependent anion-selective channel protein 2 (VDAC2) (26, 27). We estimated that VDAC2 was expressed with the same concentration as BAK in the respective cell. Only inactive BAK was modeled to bind to VDAC2. The VDAC2~BAK dimer was modeled to dissociate with an estimated backward constant $k_{backward} = 2.31x10^{-3}$ s^{-1} and a dissociation constant of 1,000 nM. The forward constant $k_{forward}$ was calculated to be $2.31x10^{-6}$ $nM^{-1}s^{-1}$ (Supplementary Table 4d).

Active BAK^* and BAX^* was modeled to homo-oligomerize up to dodecamers (Supplementary Table 5). For homo-oligomerization, a K_D of 15 nM and a decay time of the pores of 1 hour were estimated. Hexamers or larger homo-oligomers were modeled as mitochondrial pores. Once 10% of total effectors formed pores, mitochondrial outer membrane permeabilization (MOMP) was considered to be induced, in analogy with previous studies from our group (24).

To study the effect of apoptosis sensitizers, further ODEs that characterized the interaction of the BH3-mimetics ABT199, WEHI-539 and A-1210477 with the BCL2 family proteins (Supplementary Table 6) were included. Each of these drugs was modeled to bind to the anti-apoptotic proteins BCL2, BCL(X)L and MCL1. Specific dissociation constants K_D were taken from the literature (28-31), the backward constants $k_{backward}$ were fixed to reasonable values and the forward constant $k_{forward}$ resulted from $k_{backward}$ and K_D. The degradation constants k_{deg} of the antagonists and their hetero-dimers with BCL2, BCL(X)L and MCL1 were set equivalent to the degradation constants of the hetero-dimers of anti-apoptotic BCL2 proteins and the BH3-only proteins, respectively. These constants remained unchanged, as described in Lindner *el al.* (1) .

Model Input

We used two major inputs for the model. First we used absolute protein concentrations of BAK, BAX, BCL2, BCL(X)L and MCL1. Secondly, we modeled cell stress by protein production of BH3-only proteins.

We determined the concentration of BAK, BAX, BCL2, BCL(X)L and MCL1 by quantitative Western Blotting (see Figure 2 and Table 1). Since BCL2, BCL(X)L and MCL1 sustain protein degradation with half-life times of less than or equal to 5 hours (5-11), we considered a turnover for these proteins. The cell and protein specific production rate k_{prod} were calculated by multiplying the quantified (target) protein concentration by the respective degradation constant

k_{deg} of the protein (Supplementary Table 2). We modeled stable levels of BAK and BAX (protein half-life time > 22 hours (12, 13)) and therefore modeled neither protein production nor protein degradation for those proteins.

Stress induction was modeled by protein production of BIM/PUMA/NOXA (32). Unless noted otherwise, the duration of BH3-only production rate was 12 hours, between time points t = 0h and t = 12h after stress induction, and kept on a constant level. The *BH3-only stress dose η* is defined as the production rate per hour multiplied by the duration. Where indicated, the *BH3-only stress dose η* required for MOMP of these proteins was calculated by using the iterative procedure as described below. Like the *BH3-only stress dose η*, administration of ABT199, WEHI-539 and A-1210477 was modeled with a continuous increase to a specific total concentration of the respective drug over 12 hours (33, 34), between time point t = 0h and t = 12h after stress induction.

For each cell, a steady state of the protein concentration was calculated in the absence of stress. The resulting steady state was used as the initial state for the subsequent calculation in the presence of stress.

Determining of the model predicted BH3-only stress dose η that is required for MOMP

The *BH3-only protein stress dose η* (BIM/PUMA/NOXA) that is required for MOMP was determined by an iterative approximation algorithm for each cell. MOMP was considered to occur once 10% of total effectors were bound to pores (35). An initial protein stress rate (protein production per time, ν_0) of 2 μM/h (correspond to a 24 μM dose over 12 hours) was used. The initial step size for the iteration $\Delta\nu_0$ was set to the half value of the initial protein stress rate ν_0. It was then determined whether or not the model predicted MOMP in that particular cell, under the

initially used stress. If MOMP was not predicted, the used protein stress rate was increased by the step size $\Delta\nu_i$ ($\nu_{i+1} = \nu_i + \Delta\nu_i$). When the protein stress was sufficient to cause MOMP, the used protein stress was decreased by the step size $\Delta\nu_0$ ($\nu_{i+1} = \nu_i - \Delta\nu_i$). Subsequently, a new step size was set to the half of the actual value of $\Delta\nu_{i+1} = \Delta\nu_i / 2$. Calculations were repeated until the step size was smaller than 10^{-6} μM/h (correspond to a minimal dose of 1.2×10^{-5} μM over 12 hours). Finally, the BH3-only *protein stress dose* η required for MOMP was calculated by multiplying the determined protein stress ν by the duration for which the stress was modeled to be present (12 hours).

Supplementary Table Legends

Supplementary Table 1: Translation of protein interactions into Ordinary Differential Equations

Protein reactions were grouped into different classes and translated into a mathematical form according to the examples given below. Kinetic constants are described in the supplementary text. **(a)** The protein degradation rate of the protein 'C' was modeled to be proportional to the actual protein concentration. **(b)** Protein turnover of a protein 'C' was modeled to consist of constant protein production and protein degradation. **(c)** Translocation of a protein 'R' to a different compartment was modeled by a first order reaction. The translocated protein was considered as different model entity 'R*'. **(d)** Irreversible protein reaction where two proteins 'R1' and 'R2' formed a new protein 'P'. Changes of protein concentration over time were proportional to the product of reactant concentrations. **(e)** Reversible reactions of two proteins 'R1' and 'R2' forming a complex 'R1~R2'. Change of protein concentration was given by balancing forward and backward reactions.

Supplementary Table 2: Pseudo-reactions for degradation and degradation rates as used in the model

Proteins and protein complexes that were modeled to be subjected to degradation are depicted. Once stress was induced the half-life time of MCL1 was reduced from 45 (8, 10) to 17 minutes (11). We modeled stable levels of BAK and BAX (protein half-life time > 22 hours (12, 13)) and therefore modeled protein neither production nor degradation for those proteins. We considered for BCL(X)L the same half-life time as for BCL2

(300 minutes) due to the similar amino acid sequence of BCL2 and BCL(X)L (EMBOSS Needle alignment, similarity 53%). Half-life times of most complexes of anti- and pro-apoptotic proteins were modeled to be the same as the half-life time of the BCL2~tBID / BCL(X)L~tBID complex. The degradation constants k_{deg} were calculated from the half life time by the following equation: $k_{\deg} = \ln(2)/(60 * t_{1/2})$

Supplementary Table 3: Pseudo-reactions and kinetics for inhibition of BH3-only proteins and effectors BAK and BAX by anti-apoptotic proteins

Pseudo reactions and kinetic constants for binding of **(a)** BCL2, **(b)** BCL(X)L, **(c)** MCL1 to BH3-only proteins, and BAK and BAX are given. Dissociation constants K_D were taken from literature as indicated (14). Whenever no binding was reported, a K_D of 10,000 nM was modeled. Wherever indicated, the backward constant $k_{backward}$ was taken from (14) and otherwise fixed as stated in the text. No K_D for the binding of BCL2 to tBID was reported in literature. We modeled a K_D of 4 nM with respect to the assumption that the ratio of the K_D of the binding of BCL2 to BID (66 nM (18)) and BCL2 to tBID is about the same as the ratio of the K_D binding of BCL(X)L to BID (448 nM (34)) and BCL(X)L to tBID (27.2 nM (21)). Forward constants were obtained from the backward constants $k_{backward}$ and from dissociation constants K_D, by $k_{forward} = k_{backward} / K_D$

Supplementary Table 4: BAK and BAX activation and BAK inhibition by VDAC2

Activation of BAK and BAX by BIM, PUMA, tBID and by the tBID chimeras $tBID^{BAK}$ and $tBID^{BAX}$ is given. **(a)** In a first step, inactive cytosolic BAX and inactive BAK were modeled to build hetero-dimers with their respective activators. K_D values and forward/backward reaction constants were modeled as below. **(b)** Once the hetero-dimer was formed, the activation and instant dissociation between BAK and BAX and the respective

activators were modeled with a half-life time of 1/10 minute. **(c)** Activated cytosolic BAX was modeled to translocate with a half-life time 1/10 minute ($k = 1.16x10^{-1}1s^{-1}$) to the mitochondrial membrane. **(d)** Inactive BAK was modeled to be able to bind to VDAC2.

Supplementary Table 5: Effector homo-oligomerization

Activated BAK and BAX were modeled to homo-oligomerize in the mitochondrial outer membrane. Effectors were able to build homo-dimers which further were modeled to build larger homo-oligomers. Independent of the complex size, the same dissociation constants and kinetic constants were used. Homo-oligomers larger or equal than hexamers were considered as pores. Only homo-oligomers smaller than or equal to dodecamers were taken into account. Size of effector oligomers are indicated by superscripts.

Supplementary Table 6: Modeling apoptosis sensitizers ABT199, WEHI-539 and A-1210477.

Interaction kinetics of the apoptosis sensitizers ABT199, WEHi-539 and A-1210477 with BCL2 proteins were included into the model. **(a)** Binding of both sensitizers to pro-apoptotic BCL2 protein according to K_D values of the literature **(b)** degradation of selective BCL2 antagonists and their hetero-dimers with anti-apoptotic proteins was estimated.

Supplementary Tables

Supplementary Table 1

**a**	**protein and protein complex degradation**
$C \xrightarrow{k_{\deg}}$	to $\frac{d[C]}{dt} = -k_{\deg} * [C]$ whereby $\lim_{t \to \infty} [C] = \frac{k_{prod}}{k_{\deg}}$

**b**	**protein turnover**
$\xrightarrow{k_{prod}} C \; k_{\deg}$	$\frac{d[C]}{dt} = k_{prod} - k_{\deg} * [C]$

**c**	**protein translocation**
$R \xrightarrow{k} R*$	to $\frac{d[R]}{dt} = -k * [R]$ $\frac{d[R*]}{dt} = k * [R]$

**d**	**irreversible protein reaction**
$R_1 + R_2 \xrightarrow{k} P$	to $\frac{d[R_1]}{dt} = \frac{d[R_2]}{dt} = -k * [R_1] * [R_2]$ $\frac{d[P]}{dt} = k * [R_1] * [R_2]$

**e**	**reversible protein reaction (association and dissociation)**
$R_1 + R_2 \underset{k_{backward}}{\overset{k_{forward}}{\longleftrightarrow}} R_1 \sim R_2$	to $\frac{d[R_1]}{dt} = \frac{d[R_2]}{dt} = k_{backward} * [R_1 \sim R_2] - k_{forward} * [R_1] * [R_2]$ $\frac{d[R_1 \sim R_2]}{dt} = k_{forward} * [R_1] * [R_2] - k_{backward} * [R_1 \sim R_2]$ whereby $K_D = \frac{k_{backward}}{k_{forward}}$

Supplementary Table 2

Biochemical reaction		k_{deg} [s^{-1}]	$t_{1/2}$ [min]	
BCL2	→	3.85E-05	300	(6)
BCL(X)L	→	3.85E-05	300	
MCL1	→	2.57E-04	45	(8, 10)
MCL1	→	6.80E-04	17	(11)
BIM	→	4.81E-05	240	(6)
tBID	→	1.54E-05	75	(5)
PUMA	→	5.66E-05	204	(7)
NOXA	→	1.93E-04	60	(9)
BCL2 ~ BIM	→	1.54E-05	75	
BCL(X)L ~ BIM	→	1.54E-05	75	
MCL1 ~ BIM	→	7.70E-05	150	(2)
BCL2 ~ tBID	→	1.54E-05	75	(5)
BCL(X)L ~ tBID	→	1.54E-05	75	(5)
MCL1 ~ tBID	→	1.54E-05	75	
BCL2 ~ PUMA	→	1.54E-05	75	
BCL(X)L ~ PUMA	→	1.54E-05	75	
MCL1 ~ PUMA	→	7.70E-05	150	(3)
BCL2 ~ NOXA	→	1.54E-05	75	
BCL(X)L ~ NOXA	→	1.54E-05	75	
MCL1 ~ NOXA	→	2.57E-04	45	(4)

Supplementary Table 3

**a**	**Biochemical reaction**		$k_{forward}$ [$nM^{-1}s^{-1}$]	$k_{backward}$ [s^{-1}]	K_D [nM]	
BCL2 + BIM	↔	BCL2 ~ BIM	3.00E-05	1.40E-04	4.5	(14)
BCL2 + tBID	↔	BCL2 ~ tBID	3.50E-05	1.40E-04	4.0	
BCL2 + PUMA	↔	BCL2 ~ PUMA	7.78E-06	1.40E-04	18.0	(18)
BCL2 + NOXA	↔	BCL2 ~ NOXA	7.29E-07	1.40E-04	192.0	(18)
BCL2 + BAK	↔	BCL2 ~ BAK	1.40E-08	1.40E-04	10,000	(4)
BCL2 + BAX	↔	BCL2 ~ BAX	9.33E-06	1.40E-04	15.0	(36)

**b**	**Biochemical reaction**		$k_{forward}$ [$nM^{-1}s^{-1}$]	$k_{backward}$ [s^{-1}]	K_D [nM]	
BCL(X)L + BIM	↔	BCL(X)L ~ BIM	5.50E-04	4.40E-04	0.8	(14)
BCL(X)L + tBID	↔	BCL(X)L ~ tBID	1.62E-05	4.40E-04	27.2	(21)
BCL(X)L + PUMA	↔	BCL(X)L ~ PUMA	8.63E-05	4.40E-04	5.1	(19)
BCL(X)L + NOXA	↔	BCL(X)L ~ NOXA	4.40E-08	4.40E-04	10,000	(37)
BCL(X)L + BAK	↔	BCL(X)L ~ BAK	5.50E-06	4.40E-04	80.0	(15)
BCL(X)L + BAX	↔	BCL(X)L ~ BAX	5.18E-07	4.40E-04	850.0	(20)

**c**	**Biochemical reaction**		$k_{forward}$ [$nM^{-1}s^{-1}$]	$k_{backward}$ [s^{-1}]	K_D [nM]	
MCL1 + BIM	↔	MCL1 ~ BIM	1.30E-03	2.60E-04	0.2	(14)
MCL1 + tBID	↔	MCL1 ~ tBID	2.63E-05	2.60E-04	9.9	(16)
MCL1 + PUMA	↔	MCL1 ~ PUMA	1.37E-04	2.60E-04	1.9	(16)
MCL1 + NOXA	↔	MCL1 ~ NOXA	6.58E-06	2.60E-04	39.9	(16)
MCL1 + BAK	↔	MCL1 ~ BAK	3.25E-05	2.60E-04	8.0	(4, 15)
MCL1 + BAX	↔	MCL1 ~ BAX	2.60E-08	2.60E-04	10,000	(17)

Supplementary Table 4

**a**	**Biochemical reaction**		**$k_{forward}$ [$nM^{-1}s^{-1}$]**	**$k_{backward}$ [s^{-1}]**	**K_D [nM]**
Bax_c + Activator	↔	Bax_c ~ Activator	2.57E-06	2.57E-04	100.0
Bak_m + Activator	↔	Bak_m ~ Activator	2.57E-06	2.57E-04	100.0

**b**	**Biochemical reaction**		**k [s^{-1}]**	**$t_{1/2}$ [min]**
Bak_m ~ Activator	→	Bak^*_m + Activator	1.16E-01	0.1
Bax_c ~ Activator	→	Bax^*_c + Activator	1.16E-01	0.1

**c**	**Biochemical reaction**		**k [s^{-1}]**	**$t_{1/2}$ [min]**
Bax^*_c	→	Bax^*_m	1.16E-01	0.1

**d**	**Biochemical reaction**		**$k_{forward}$ [$nM^{-1}s^{-1}$]**	**$k_{backward}$ [s^{-1}]**	**K_D [nM]**
Bak_m + VDAC2	↔	Bak_m ~ VDAC2	2.31E-06	2.31E-03	1,000.0

Supplementary Table 5

Biochemical reaction			$k_{forward}$ [$nM^{-1}s^{-1}$]	$k_{backward}$ [s^{-1}]	K_D [nM]
$Effector^{*}_{m}$ + $Effector^{*}_{m}$	↔	$Effector^{2}_{m}$	1.28E-05	1.93E-04	15
$Effector^{2}_{m}$ + $Effector^{2}_{m}$	↔	$Effector^{4}_{m}$	1.28E-05	1.93E-04	15
$Effector^{2}_{m}$ + $Effector^{4}_{m}$	↔	$Effector^{6}_{m}$	1.28E-05	1.93E-04	15
$Effector^{2}_{m}$ + $Effector^{6}_{m}$	↔	$Effector^{8}_{m}$	1.28E-05	1.93E-04	15
$Effector^{4}_{m}$ + $Effector^{4}_{m}$	↔	$Effector^{8}_{m}$	1.28E-05	1.93E-04	15
$Effector^{4}_{m}$ + $Effector^{7}_{m}$	↔	$Effector^{10}_{m}$	1.28E-05	1.93E-04	15
$Effector^{2}_{m}$ + $Effector^{8}_{m}$	↔	$Effector^{10}_{m}$	1.28E-05	1.93E-04	15
$Effector^{6}_{m}$ + $Effector^{6}_{m}$	↔	$Effector^{12}_{m}$	1.28E-05	1.93E-04	15
$Effector^{4}_{m}$ + $Effector^{8}_{m}$	↔	$Effector^{12}_{m}$	1.28E-05	1.93E-04	15
$Effector^{2}_{m}$ + $Effector^{10}_{m}$	↔	$Effector^{12}_{m}$	1.28E-05	1.93E-04	15

Supplementary Table 6

a

Biochemical reaction			$k_{forward}$ [$nM^{-1}s^{-1}$]	$k_{backward}$ [s^{-1}]	K_D [nM]	
ABT199 + BCL2	↔	ABT199 ~ BCL2	0.0193	1.93E-04	0.010	(31)
ABT199 + BCL(X)L	↔	ABT199 ~ BCL(X)L	4.02E-06	1.93E-04	48.0	(31)
ABT199 + MCL1	↔	ABT199 ~ MCL1	4.34E-07	1.93E-04	444.0	(31)
WEHI-539 + BCL2	↔	WEHI-539 ~ BCL2	2.57E-07	1.93E-04	750	(29)
WEHI-539 + BCL(X)L	↔	WEHI-539 ~ BCL(X)L	0.000137	1.93E-04	1.4	(29)
WEHI-539 + MCL1	↔	WEHI-539 ~ MCL1	3.50E-07	1.93E-04	550	(29)
A-1210477 + BCL2	↔	A-1210477 ~ BCL2	1.46E-06	1.93E-04	132	(30)
A-1210477 + BCL(X)L	↔	A-1210477 ~ BCL(X)L	2.92E-07	1.93E-04	660	(30)
A-1210477 + MCL1	↔	A-1210477 ~ MCL1	0.000425	1.93E-04	0.454	(30)

b

Biochemical reaction		k_{deg} [s^{-1}]	$t_{1/2}$ [min]
Antagonist	→	9.63E-05	120
Antagonist ~ BCL2	→	1.54E-04	75
Antagonist ~ BCL(X)L	→	1.54E-04	75
Antagonist ~ MCL1	→	7.70E-05	150

Supplement References

1. Lindner AU, Concannon CG, Boukes GJ, Cannon MD, Llambi F, Ryan D, et al. Systems analysis of BCL2 protein family interactions establishes a model to predict responses to chemotherapy. Cancer research. 2013;73(2):519-28.

2. Czabotar PE, Lee EF, van Delft MF, Day CL, Smith BJ, Huang DCS, et al. Structural insights into the degradation of Mcl-1 induced by BH3 domains. Proceedings of the National Academy of Sciences. 2007;104(15):6217-22.

3. Mei Y, Du W, Yang Y, Wu M. Puma*Mcl-1 interaction is not sufficient to prevent rapid degradation of Mcl-1. Oncogene. 2005;24(48):7224-37.

4. Willis SN, Chen L, Dewson G, Wei A, Naik E, Fletcher JI, et al. Proapoptotic Bak is sequestered by Mcl-1 and Bcl-xL, but not Bcl-2, until displaced by BH3-only proteins. Genes & development. 2005;19(11):1294-305.

5. Breitschopf K, Zeiher AM, Dimmeler S. Ubiquitin-mediated Degradation of the Proapoptotic Active Form of Bid. Journal of Biological Chemistry. 2000;275(28):21648-52.

6. Dimmeler S, Breitschopf K, Haendeler J, Zeiher AM. Dephosphorylation Targets Bcl-2 for Ubiquitin-dependent Degradation: A Link between the Apoptosome and the Proteasome Pathway. The Journal of experimental medicine. 1999;189(11):1815-22.

7. Fricker M, O'Prey J, Tolkovsky AM, Ryan KM. Phosphorylation of Puma modulates its apoptotic function by regulating protein stability. Cell Death and Disease. 2010;1(7):e59.

8. Kubota Y, Kinoshita K, Suetomi K, Fujimori A, Takahashi S. Mcl-1 Depletion in Apoptosis Elicited by Ionizing Radiation in Peritoneal Resident Macrophages of C3H Mice. The Journal of Immunology. 2007;178(5):2923-31.

9. Mei Y, Xie C, Xie W, Tian X, Li M, Wu M. Noxa/Mcl-1 Balance Regulates Susceptibility of Cells to Camptothecin-Induced Apoptosis. Neoplasia (New York, NY). 2007;9(10):871-81.

10. Nijhawan D, Fang M, Traer E, Zhong Q, Gao W, Du F, et al. Elimination of Mcl-1 is required for the initiation of apoptosis following ultraviolet irradiation. Genes & development. 2003;17(12):1475-86.

11. Schwickart M, Huang X, Lill JR, Liu J, Ferrando R, French DM, et al. Deubiquitinase USP9X stabilizes MCL1 and promotes tumour cell survival. Nature. 2009;463(7277):103-7.

12. Xu S, Peng G, Wang Y, Fang S, Karbowski M. The AAA-ATPase p97 is essential for outer mitochondrial membrane protein turnover. Molecular biology of the cell. 2010;22(3):291-300.

13. Moulding DA, Akgul C, Derouet M, White MR, Edwards SW. BCL-2 family expression in human neutrophils during delayed and accelerated apoptosis. Journal of leukocyte biology. 2001;70(5):783-92.

14. Chen L, Willis SN, Wei A, Smith BJ, Fletcher JI, Hinds MG, et al. Differential Targeting of Prosurvival Bcl-2 Proteins by Their BH3-Only Ligands Allows Complementary Apoptotic Function. Molecular cell. 2005;17(3):393-403.

15. Dai H, Meng XW, Lee S-H, Schneider PA, Kaufmann SH. Context-dependent Bcl-2/Bak Interactions Regulate Lymphoid Cell Apoptosis. Journal of Biological Chemistry. 2009;284(27):18311-22.

16. Day CL, Smits C, Fan FC, Lee EF, Fairlie WD, Hinds MG. Structure of the BH3 Domains from the p53-Inducible BH3-Only Proteins Noxa and Puma in Complex with Mcl-1. Journal of Molecular Biology. 2008;380(5):958-71.

17. Germain M, Milburn J, Duronio V. MCL-1 Inhibits BAX in the Absence of MCL-1/BAX Interaction. Journal of Biological Chemistry. 2007;283(10):6384-92.

18. Jette CA, Flanagan AM, Ryan J, Pyati UJ, Carbonneau S, Stewart RA, et al. BIM and other BCL-2 family proteins exhibit cross-species conservation of function between zebrafish and mammals. Cell death and differentiation. 2008;15(6):1063-72.

19. Lama D, Sankararamakrishnan R. Anti-apoptotic Bcl-XL protein in complex with BH3 peptides of pro-apoptotic Bak, Bad, and Bim proteins: Comparative molecular dynamics simulations. Proteins: Structure, Function, and Bioinformatics. 2008;73(2):492-514.

20. Tan Y-J, Ting AE. Non-ionic detergent affects the conformation of a functionally active mutant of Bcl-XL. Protein Engineering, Design and Selection. 2000;13(12):887-92.

21. Yao Y, Bobkov AA, Plesniak LA, Marassi FM. Mapping the Interaction of Pro-Apoptotic tBID with Pro-Survival BCL-XL. Biochemistry. 2009;48(36):8704-11.

22. Vogel S, Raulf N, Bregenhorn S, Biniossek ML, Maurer U, Czabotar P, et al. Cytosolic Bax: does it require binding proteins to keep its pro-apoptotic activity in check? The Journal of biological chemistry. 2012;287(12):9112-27.

23. Dai H, Smith A, Meng XW, Schneider PA, Pang Y-P, Kaufmann SH. Transient binding of an activator BH3 domain to the Bak BH3-binding groove initiates Bak oligomerization. The Journal of cell biology. 2011;194(1):39-48.

24. Düssmann H, Rehm M, Concannon CG, Anguissola S, Würstle M, Kacmar S, et al. Single-cell quantification of Bax activation and mathematical modelling suggest pore formation on minimal mitochondrial Bax accumulation. Cell death and differentiation. 2009;17(2):278-90.

25. Korsmeyer SJ, Wei MC, Saito M, Weiler S, Oh KJ, Schlesinger PH. Pro-apoptotic cascade activates BID, which oligomerizes BAK or BAX into pores that result in the release of cytochrome c. Cell death and differentiation. 2000;7(12):1166-73.

26. Cheng EH, Sheiko TV, Fisher JK, Craigen WJ, Korsmeyer SJ. VDAC2 inhibits BAK activation and mitochondrial apoptosis. Science (New York, NY). 2003;301(5632):513-7.

27. Lazarou M, Stojanovski D, Frazier AE, Kotevski A, Dewson G, Craigen WJ, et al. Inhibition of Bak Activation by VDAC2 Is Dependent on the Bak Transmembrane Anchor. Journal of Biological Chemistry. 2010;285(47):36876-83.

28. Lessene G, Czabotar PE, Colman PM. BCL-2 family antagonists for cancer therapy. Nature Reviews Drug Discovery. 2008;7(12):989-1000.

29. Lessene G, Czabotar PE, Sleebs BE, Zobel K, Lowes KN, Adams JM, et al. Structure-guided design of a selective BCL-XL inhibitor. Nat Chem Biol. 2013;9(6):390-7.

30. Leverson JD, Zhang H, Chen J, Tahir SK, Phillips DC, Xue J, et al. Potent and selective small-molecule MCL-1 inhibitors demonstrate on-target cancer cell killing activity

as single agents and in combination with ABT-263 (navitoclax). Cell death & disease. 2015;6(1):e1590.

31. Souers AJ, Leverson JD, Boghaert ER, Ackler SL, Catron ND, Chen J, et al. ABT-199, a potent and selective BCL-2 inhibitor, achieves antitumor activity while sparing platelets. Nature medicine. 2013;19(2):202-8.

32. Mariadason JM, Arango D, Shi Q, Wilson AJ, Corner GA, Nicholas C, et al. Gene expression profiling-based prediction of response of colon carcinoma cells to 5-fluorouracil and camptothecin. Cancer research. 2003;63(24):8791-812.

33. DiMasi JA, Feldman L, Seckler A, Wilson A. Trends in Risks Associated With New Drug Development: Success Rates for Investigational Drugs. Clinical Pharmacology & Therapeutics. 2010;87(3):272-7.

34. Rubin EH, Gilliland DG. Drug development and clinical trials—the path to an approved cancer drug. Nature Reviews Clinical Oncology. 2012;9(4):215-22.

35. Huber HJ, Duessmann H, Wenus J, Kilbride SM, Prehn JHM. Mathematical modelling of the mitochondrial apoptosis pathway. Biochimica et Biophysica Acta (BBA) - Molecular Cell Research. 2011;1813(4):608-15.

36. Ku B, Liang C, Jung JU, Oh B-H. Evidence that inhibition of BAX activation by BCL-2 involves its tight and preferential interaction with the BH3 domain of BAX. Cell Research. 2010;21(4):627-41.

37. Mérino D, Giam M, Hughes PD, Siggs OM, Heger K, O'Reilly LA, et al. The role of BH3-only protein Bim extends beyond inhibiting Bcl-2–like prosurvival proteins. The Journal of cell biology. 2009;186(3):355-62.
